# Dealing with Desirable Difficulties: Supporting Students to Accept, Reduce, or Silence Effort

**DOI:** 10.1007/s40670-023-01911-y

**Published:** 2023-10-13

**Authors:** Anique B. H. de Bruin

**Affiliations:** Department of Educational Development and Research, School of Health Professions Education, Maastricht University, Universiteitssingel 60, Postbus 616, 6200 MD Maastricht, The Netherlands

**Keywords:** Self-regulated learning, Desirable difficulties, Perceived effort, Perceived learning, Interventions

## Abstract

In this writing, I summarize the insights from my keynote lecture at the annual 2023 IAMSE Meeting in Cancún, Mexico, titled “Effort is the new smart. Supporting students in the self-regulated use of desirable difficulties.” I explain how self-regulated learning is challenging for many students in higher education and even more so under learning conditions that create desirable difficulties: conditions that foster long-term learning and transfer of knowledge and skills, but that are generally more effortful to engage in. I describe how the intricate relation between perceived effort and perceived learning determines students’ use of desirable difficulties. Finally, I outline promising interventional approaches academic teachers can employ to support students to seek out and engage in desirable difficulties.

A student who is completely surprised by failing an exam, a student who sets learning goals that are out of reach, or a student who procrastinates learning until mere days before the exam: anyone who has ever taught in higher education will be familiar with one or more of these scenarios. As higher education teachers, we are experts in recognizing students’ struggles with self-regulating their learning; that is, students’ planning, reflection, and evaluation of their learning by taking control of the cognitive, metacognitive, motivational, and emotional aspects of learning [1]. Now, one may ask whether poor self-regulated learning skills are truly problematic in higher education, because many of these students go on to graduate with a degree. Yes, is the definite answer. Because ample research shows that self-regulation skills are not only vital to thrive in academia [2], but are also predictors of adaptation to professional life, of well-being, and of general life satisfaction [3]: Having strong self-regulation skills appears to make you a happier person. And after all, the aim of higher education reaches beyond obtaining the degree. Its aim is first and foremost to prepare students for a (professional) future and empower them to flexibly adapt to any minor or major decision they will face.

Self-regulation of learning becomes even more challenging when learners face situations where learning costs (high) effort and learning effects are hidden or emerge after a delay. These conditions of learning create what are termed “desirable difficulties [4]. Desirable difficulties optimize effects on students’ long-term learning even though it may hinder or harm immediate performance. Creating desirable difficulties is what we as academic teachers are continuously after: challenging our students to engage in effortful learning that will increase long-term knowledge and skills that are transferable to novel contexts. Desirable difficulties come in various shapes and sizes and include actively retrieving information from memory by taking quizzes (vs merely rereading the textbook) [5] or mixing learning of exemplars of to-be-learned categories, such as lung diseases on x-rays (vs lumping all exemplars from one category before continuing to the next) — for a review of effective learning techniques that create desirable difficulties see [6–8]. Yet, the high effort these desirable difficulties evoke combined with the often delayed effects on learning prevents students from spontaneously engaging in desirable difficulties during self-study [9]. Students often interpret the high effort and the absence of immediate learning effects as indicators of poor learning [10], which results in a paradox between students’ experienced learning and their actual learning: The effort that is associated with desirable difficulties is conducive to learning, not disrupting it [11]. Students’ limited engagement in desirable difficulties potentially threatens their academic development: Many students prefer learning conditions that do not promote long-term learning (even though it may be sufficient to pass their exams) because they erroneously believe these circumstances to be superior, or at the very least sufficiently effective, and less costly in terms of effort. If our aim is to foster students’ long-term knowledge and skills and to prepare them optimally for a balanced professional and personal life, then supporting them to engage in, rather than avoid, desirable difficulties is a major goal for us as academic teachers.

In the remainder of this writing, I will describe some promising interventional approaches to support students to engage in desirable difficulties. But first, I will outline how perceived effort and perceived learning are intricately related to use of desirable difficulties to set the stage for the interventional approaches. These insights were previously published in more detail in Educational Psychology Review [12] and are part of the Start and Stick to Desirable Difficulties (S2D2) Framework.

## The Intricate Relation between Perceived Effort, Perceived Learning, and Use of Desirable Difficulties

**Fig. 1:**
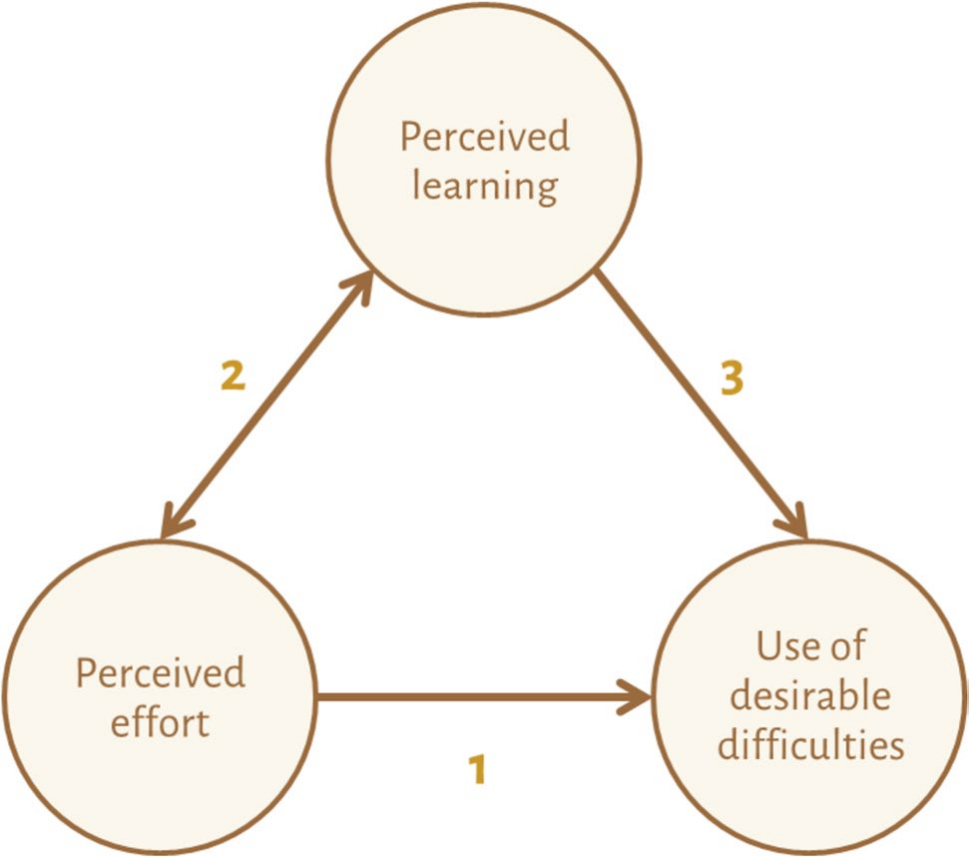
The possible relations between perceived effort, perceived learning, and learners’ engagement in desirable difficulties. Figure is reused without adaptation from [12], under the Creative Commons License (https://creativecommons.org/licenses/by/4.0/)

As the experienced-learning-versus-actual-learning paradox shows, students’ perceptions of effort and perceptions of learning are intricately related to their willingness to use desirable difficulties (see Fig. 1). When and how the three relations described in Fig. 1 apply varies within and across learners, learning tasks, and contexts. Starting with the relation between perceived effort and use of desirable difficulties (Relation 1 in Fig. 1), many learners generally interpret putting in effort as costly or aversive [13, 14], and despite potential learning gains, they will act to minimize effort investment. Research has shown that one reason why effort is experienced as costly is because it is weighed against alternative possibilities [15]: Having an easier alternative (e.g., rereading) available increases the perceived costs of the more challenging, yet beneficial strategy (e.g., retrieval practice). As for the second relation in Fig. 1, research revealed that students’ perceived effort on a task is often linked to the perceived level of learning of the task. A meta-analysis showed a negative correlation of − 0.355 between students’ effort ratings and their monitoring judgments of learning [16]. This finding indicates that students indeed tend to interpret high effort as an indication of poor learning, potentially leading to underuse of desirable difficulties which are experienced as effortful. The finding also shows that students tend towards a data-driven interpretation of effort: The task experience of high effort is what drives their conclusion about low perceived learning [17]. Instead, use of desirable difficulties can be increased when students take a goal-driven perspective towards effort. In this perspective, students have set a particular learning goal and are conscious of the fact that achieving the goal will cost (high) effort. Consequently, experiencing (high) effort is interpreted as a positive indicator of learning and goal progress.

Finally, there are circumstances when relation 3 (see Fig. 1) applies: Students are highly motivated to reach a certain goal and are not hindered by negative perceptions of effort. In fact, they do not or hardly notice effort when engaging in the task; perceptions of effort are silenced. This extremely-goal-driven situation [12] is at play when a task is highly important to a student, for instance, because of the high stakes consequences or because a huge deadline is nearing. Pulling all-nighters without noticing tiredness is one example of such a situation [18]. Under extremely-goal-driven circumstances, students may be more willing to engage in desirable difficulties. At the same time, creating these circumstances is controversial, as students’ well-being is likely to suffer at some point.

## Changing Perceptions of Effort to Increase Use of Desirable Difficulties

In what is described above, I have tried to explain how erroneous perceptions of effort are at the heart of limited use of desirable difficulties by students in higher education. Fortunately, empirical evidence and theorization on how to intervene and increase use of desirable difficulties is growing and allows for some general recommendations that can be translated to specific contexts. If these perceptions of effort are at the core, then tackling them is key. Given that the difficulties experienced under these conditions are inherent to the learning activity, especially at the start, little can be done to objectively reduce this difficulty. But how students *experience* the difficulty is amenable to change, and this change can affect their behavioral inclinations to use desirable difficulties [19]. Specifically, students can be supported to (1) accept, (2) reduce, or, in rare circumstances, (3) silence their perceptions of (high) effort. Given the readership of this journal, I will emphasize those interventional approaches that are at the hands of teachers to initiate and support. For more information on learner-initiated approaches and other teacher-initiated approaches, see de Bruin et al. [12].

### Helping Students to Accept Effort in Desirable Difficulties

When it comes to helping students to *accept* the high effort that desirable difficulties entail, it is vital to educate them explicitly about the concept of desirable difficulties, the experienced-learning-versus-actual-learning paradox, and the (lack of) effectiveness of different learning strategies. This information is mostly unknown to students [9], and while simply having the information is not sufficient to elicit behavioral change, sharing it and ensuring students’ understanding of it is essential to lay the basis for their willingness to explore the self-regulated use of desirable difficulties. The importance of explicit explanation by teachers is needed because of the non-intuitive character of some of the information (e.g., rereading feels more effective than self-testing, but it is not) and the complexity of some of the information (e.g., applying a schedule of interleaved practice). Teachers may hold the belief that students thrive when they are provided the space and time to find out their own way of studying when they enter higher education. However, withholding scientific information about desirable difficulties and effective learning strategies from our students will sell them short and creates a risk of feeding inequality between students when some students gain access to the information and others can’t. I therefore plea for incorporation of knowledge on the “science of effective learning” at an early phase in all bachelor programs to ensure a fair start to all students. This can be done within the first course by the lecturer, in mentoring or tutoring programs, or in separate learning strategy programs. For an example of an implementation, see the Study Smart program developed and researched at Maastricht University (www.studysmartpbl.com) [20]. Note that such information is ideally combined with actual exploration of use of the desirable difficulties, to have students experience (changes in) effort and gain confidence in the use of desirable difficulties under the supervision of a teacher. Actual practice with the desirable difficulties is also crucial to experience their learning benefits: Only after repeated use and at a delay after learning will these benefits become salient.

### Helping Students to Reduce or Silence Effort in Desirable Difficulties

Interventional approaches to reduce students’ perceptions of effort align with those that silence perceptions of effort. These approaches are aimed at having students experience the desirable difficulties across multiple learning opportunities for a period of time [19]. When engaging with the desirable difficulties, perceived effort will mostly be high in initial use, when learners are unfamiliar with the learning activity, which causes higher mental load. Extending the period of use of desirable difficulties, and supervising the use of desirable difficulties by reflecting on students’ experiences and possible misconceptions, will lead to an increase in students’ perceptions of the effectiveness of the desirable difficulties (also termed their perceived learning). This increase in perceived learning tends to go hand in hand with a reduction in perceived effort [16]. The extended experience increases students’ experiences of “fluency” or smoothness of learning and reduces the sense of struggle that is associated with desirable difficulties during initial use. Additionally, manipulating the (perceived) length of a learning activity by segmenting it into smaller chunks can be an effective manner to reduce perceived effort [21]. In certain circumstances, when students have high (intrinsic or extrinsic) motivation, they do not experience the effort (at all) and find themselves in a state of flow [22]. For an academic teacher, getting their students to reach this state of silenced perceived effort is not, but educating their students about conditions that enable flow is an important first step.

## Conclusion

Academic teachers play a vital role in supporting students to seek out and persist on learning activities that maximally challenge them and that do not only improve their long-term learning but also enable transfer of knowledge and skills to novel contexts later in their education or in their professional life. The high effort that these learning activities bring poses a continuous challenge that teachers can support their students to learn to deal with. In this paper, which summarizes the main messages of my keynote lecture at the 2023 IAMSE conference, I addressed this issue further and provided possible ways forward in supporting students to deal with this issue. I explained how (a combination of) theory-based interventional approaches that aim to educate students about (a) the importance of desirable difficulties, and (b) the science of (in)effective learning strategies, and experiential approaches that aim to let students experience the use of desirable difficulties have been proven promising to mend students’ misconceptions and increase their engagement in desirable difficulties. Accepting, reducing, or silencing effort is considered key in these interventional approaches. These insights provide grounds for further scientific endeavors, such as unraveling how teacher support can reduce experienced effort. At the same time, the insights pave the way for implementation in educational practice through incorporation in teaching practice or through learning strategy programs such as Study Smart.

## Data Availability

Not applicable.
